# User Experiences of the Cue2walk Smart Cueing Device for Freezing of Gait in People with Parkinson’s Disease

**DOI:** 10.3390/s25154702

**Published:** 2025-07-30

**Authors:** Matthijs van der Laan, Marc B. Rietberg, Martijn van der Ent, Floor Waardenburg, Vincent de Groot, Jorik Nonnekes, Erwin E. H. van Wegen

**Affiliations:** 1 Department of Rehabilitation Medicine, Amsterdam University Medical Center, 1081 HZ Amsterdam, The Netherlands; m.vanderlaan1@amsterdamumc.nl (M.v.d.L.); m.rietberg@amsterdamumc.nl (M.B.R.); v.degroot@amsterdamumc.nl (V.d.G.); 2 Amsterdam Movement Sciences, Rehabilitation & Development, 1081 HZ Amsterdam, The Netherlands; 3 Cue2Walk International B.V., 2521 AL The Hague, The Netherlands; martijn@cue2walk.nl (M.v.d.E.); floor@cue2walk.nl (F.W.); 4 Radboud University Medical Centre, Donders Institute for Brain, Cognition and Behaviour, Department of Rehabilitation, Centre of Expertise for Parkinson & Movement Disorders, 6525 GA Nijmegen, The Netherlands; jorik.nonnekes@radboudumc.nl; 5 Department of Rehabilitation, Sint Maartenskliniek, 6574 NA Nijmegen, The Netherlands

**Keywords:** Parkinson’s disease, freezing of gait, freezing of gait detection, cueing, smart cueing, on-demand, self-activation, wearable sensors, user experiences

## Abstract

Freezing of gait (FoG) impairs mobility and daily functioning and increases the risk of falls, leading to a reduced quality of life (QoL) in people with Parkinson’s disease (PD). The Cue2walk, a wearable smart cueing device, can detect FoG and hereupon provides rhythmic cues to help people with PD manage FoG in daily life. This study investigated the user experiences and device usage of the Cue2walk, and its impact on health-related QoL, FoG and daily activities. Twenty-five users of the Cue2walk were invited to fill out an online survey, which included a modified version of the EQ-5D-5L, tailored to the use of the Cue2walk, and its scale for health-related QoL, three FoG-related questions, and a question about customer satisfaction. Sixteen users of the Cue2walk completed the survey. Average device usage per day was 9 h (SD 4). Health-related QoL significantly increased from 5.2/10 (SD 1.3) to 6.2/10 (SD 1.3) (*p* = 0.005), with a large effect size (Cohen’s d = 0.83). A total of 13/16 respondents reported a positive effect on FoG duration, 12/16 on falls, and 10/16 on daily activities and self-confidence. Customer satisfaction was 7.8/10 (SD 1.7). This pilot study showed that Cue2walk usage per day is high and that 15/16 respondents experienced a variety of positive effects since using the device. To validate these findings, future studies should include a larger sample size and a more extensive set of questionnaires and physical measurements monitored over time.

## 1. Introduction

People with Parkinson’s disease (PD) often describe an episode of freezing of gait (FoG) as a transient sensation of their feet being glued to the ground and an inability to move in the desired direction. FoG is formally defined as a “brief, episodic absence or marked reduction of forward progression of the feet despite the intention to walk” [1] (p. 734). Episodes of FoG mostly arise during gait initiation, turning, and crossing narrow spaces, such as doors [2]. It is a very disabling phenomenon and impacts around 65% of people with PD in advanced stages of the disease [3]. FoG is associated with an increased risk of falls, fear of falling, and anxiety, and it is a predictor of a lower quality of life (QoL) [4,5,6,7,8].

Pharmacological treatments for FoG, such as levodopa, remain insufficiently effective, while surgical treatments, such as deep brain stimulation, are invasive, and studies show contradictory results on the efficacy [9,10,11]. This necessitates the exploration of alternative, non-pharmacological interventions. One such approach is external rhythmic cueing (e.g., a metronome), which has demonstrated potential in alleviating FoG, also in home-based settings [12], by shifting the focus of attention to gait [13,14]. Traditional implementations of external cueing typically involve either manual activation or continuously present cueing. However, manual activation may not be timely enough to address sudden FoG episodes and often requires assistance from a caregiver, which limits the independence of people with PD and places a burden on their relatives or a clinician, which limits the use of cueing in at-home situations where help is the most needed. Conversely, continuous cueing, while potentially beneficial, can be perceived as intrusive or disturbing, limiting its long-term usability.

Due to recent technological developments, external cueing can now be triggered upon the real-time detection of an episode of FoG by a wearable sensor. This type of cueing, also called ‘smart cueing’, can be used outside of the clinic and can be implemented in the daily life and own environment of people with PD. It has shown promising effects on alleviating FoG [15,16,17,18]. However, the systems used in these studies are often impractical, with multisensory setups and/or sensors at impractical locations, which limits the usability of the systems outside of the lab.

**Figure 1:**
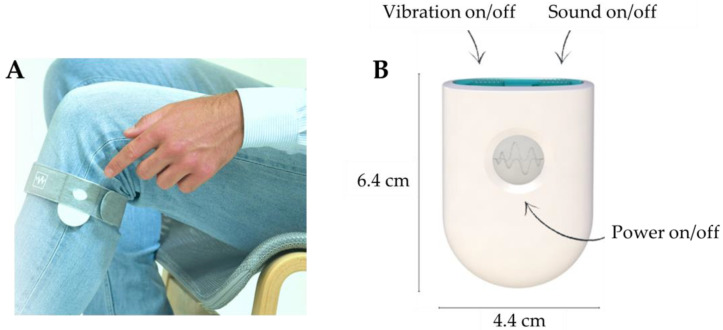
(**A**) The Cue2walk device. (**B**) The dimensions of the Cue2walk device and the function of the button and the two sliders on the device.

To address the limitations of existing smart cueing systems, a novel single-sensor smart cueing device, the Cue2walk (Cue2Walk International B.V., The Hague, The Netherlands), has been developed and is designed to be worn on the lateral side of the shank just below the knee (Figure 1). This device provides auditory and/or vibratory cues, at preference of the wearer. Cueing can be activated manually, via a button press or a hands-free motion (i.e., heel tap), or automatically by the real-time detection of an episode of FoG. Because gait kinematics during an episode of FoG are different than during normal walking, FoG can be detected in acceleration data. The Cue2walk device contains tri-axial accelerometers to collect this data, which is then processed by a combination of up to six algorithms with a sample frequency of 32$\frac{1}{3}$ Hz for the detection of FoG episodes. In a previous study, we found that the Cue2walk device showed good performance to detect FoG, with a sensitivity of 89%, a specificity of 93%, and a latency of 1.6 s [19]. By integrating real-time detection with adequate external cueing in a single-sensor device, the device aims to enhance usability and accessibility in the everyday environment. However, empirical evidence regarding its usability and effectiveness in alleviating FoG remains limited. Therefore, this pilot study aimed to systematically investigate the user experiences and device usage of the Cue2walk smart cueing device in people with PD experiencing FoG, and its impact on perceived health-related QoL, FoG, and daily activities.

## 2. Materials and Methods

### 2.1. Participant Selection

In this retrospective open-label pilot study, 25 users of the Cue2walk device were selected randomly by a digital random number generator in a database of 104 users of the device.

### 2.2. Online Survey

An email containing a link to the online survey in Google Forms (Google LLC, Mountain View, CA, USA) was distributed to the 25 users of the Cue2walk device (Cue2Walk International B.V., The Hague, The Netherlands). The survey included questions about demographics, Cue2walk device usage per day in hours, and possession of the device in months; a modified version of the EuroQol 5 Dimensions 5 Level Survey (EQ-5D-5L), including a rating on a 0–10 Visual Analogue Scale (VAS) for overall health-related QoL (comparing the current situation to before the use of the Cue2walk device), three additional FoG-related questions, and a question about customer satisfaction. See Appendix A for a detailed overview of the survey.

The questions of the EQ-5D-5L normally assess a certain domain at only one time point (i.e., the time of filling out the questionnaire) and were therefore modified in such a way that the questions concern the change in experiences since using the Cue2Walk device, e.g., ‘Have you experienced any changes in your mobility since using the Cue2Walk device?’ There were five response options for each question on the modified EQ-5D-5L and three additional questions: the impact of the use of the Cue2walk device on the domain in question was very positive (Score 2), positive (Score 1), very negative (Score −2), negative (Score −1), or the use of the Cue2walk device did not have an impact (Score 0).

In order to obtain the highest possible response rate, the respondents were not required to answer every question.

### 2.3. Data Analysis

Data analysis was performed in Microsoft Excel 365 (version 2408, Microsoft Corporation, Redmond, WA, USA) and GraphPad Prism (version 10.2.0, GraphPad Software, San Diego, CA, USA) by an independent researcher not involved in the data collection process (M.v.d.L.). Differences on the modified EQ-5D-5L VAS before using the Cue2walk device and at the moment of filling out the survey were assessed with a Student’s paired t-test. Cohen’s d was used to determine the effect size, with values of 0.2 considered a small effect, 0.5 a moderate effect, and 0.8 a large effect [20]. Missing data was interpolated by using the mean score of the other respondents on that question. Due to the ordinal nature of the data, Spearman’s rank correlations were used to determine the association between device usage per day and the various domains of the modified EQ-5D-5L, the three additional FoG-related questions, and customer satisfaction. A two-tailed *p*-value of <0.05 was considered significant.

## 3. Results

### 3.1. Participant Characteristics

From the 25 users of the Cue2walk device to whom the online survey was distributed, 17 completed it. Sixteen respondents were diagnosed with PD and one with an atypical parkinsonism (progressive supranuclear palsy). One of the respondents did not use the Cue2walk device at the time of filling out the survey. This respondent responded with a neutral answer to all the questions, but it is unknown whether this respondent did not experience any impact because he or she did not use the device or vice versa. Therefore, this respondent was excluded from further analysis. The characteristics of the remaining 16 respondents are summarized in Table 1.

### 3.2. Question Completion Rate

Across all participants, 93.75% of the questions of the modified EQ-5D-5L and the three additional FoG-related questions were answered, while 100% of the participants rated customer satisfaction and the VAS of the modified EQ-5D-5L for both before using the Cue2walk device and the moment of filling out the survey.

### 3.3. Possession of the Device and Device Usage

The participants possessed the device on average for 6 months (SD 4) and used it on average for 9 h per day (SD 4). Two participants indicated that they used the device only when they felt they needed to use it.

### 3.4. Modified EQ-5D-5L VAS Score

**Figure 2:**
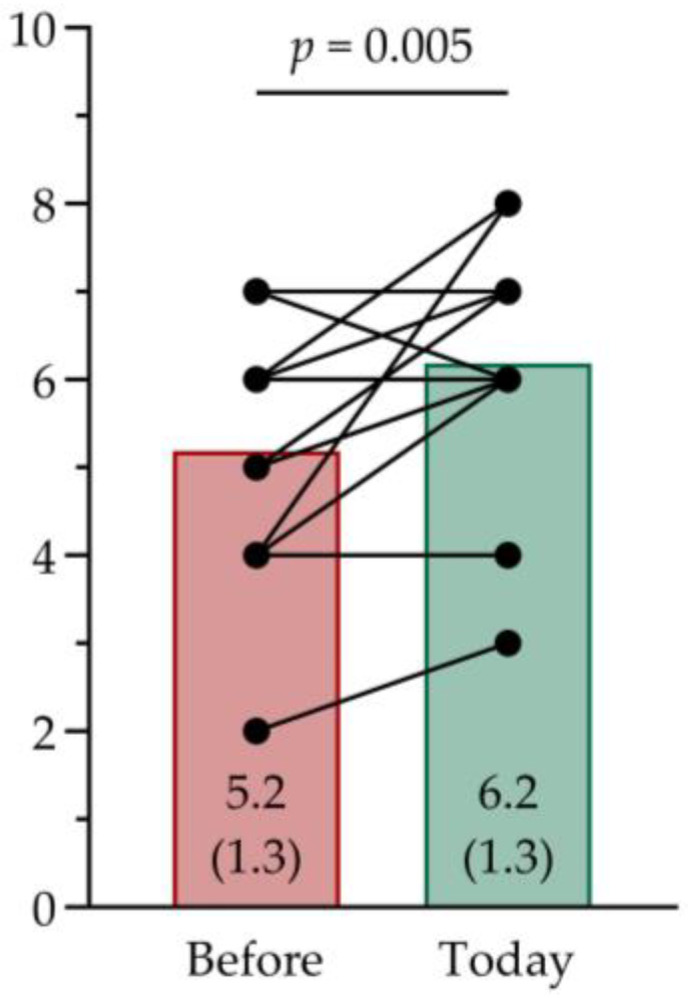
Individual and average ratings for overall health-related quality of life on the Visual Analogue Scale (VAS) of the modified EuroQol 5 Dimensions 5 Level Survey (EQ-5D-5L). A significant improvement (*p* = 0.005) was found from before the use of the Cue2walk device to the moment of filling out the survey.

The respondents rated their overall health-related QoL a 5.2 (SD 1.3) before using the Cue2walk device and a 6.2 (SD 1.3) at the moment of filling out the survey on a 0–10 VAS (Figure 2). This improvement was significant (*p* = 0.005) with a large effect size (Cohen’s d = 0.83, 95% CI [0.45, 1.48]) [20], and, moreover, a change from an insufficient rating for overall health-related QoL to a sufficient rating. One respondent reported a decreased rating for overall health-related QoL at the moment of filling out the survey compared to before using the Cue2walk device, from a 7 to a 6 on a 0–10 VAS.

### 3.5. Modified EQ-5D-5L and FoG-Related Questions

**Figure 3:**
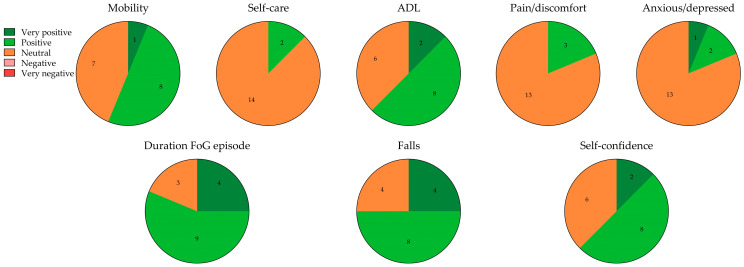
Responses of the 16 users of the Cue2walk device on the various domains of the modified EuroQol 5 Dimensions 5 Level Survey (EQ-5D-5L) (upper plot) and the FoG-related questions (lower plot). ADL, activities of daily living; FoG, freezing of gait.

The respondents experienced predominantly positive effects on various domains of the modified EQ-5D-5L and the three additional FoG-related questions since using the Cue2walk device (Figure 3). The most positive responses were given for mobility (6.25% very positive, 50% positive), activities of daily living (ADL; 12.5% very positive, 50% positive), duration of an episode of FoG (25% very positive, 56.25% positive), number of falls (25% very positive, 50% positive), and self-confidence (12.5% very positive, 50% positive). Positive effects were also reported on the domains of self-care, pain/discomfort, and anxiety/depression, but to a lesser extent than on the other domains. A total of 15 of the 16 respondents reported at least one positive effect, while 14 of the 16 respondents reported at least two positive effects. Importantly, no negative effects were reported on any of the domains.

### 3.6. Customer Satisfaction

On the question how likely they were to recommend the Cue2walk device to someone else with PD experiencing FoG, the respondents’ average rating was 7.8 (SD 1.7) on a 0–10 scale. One respondent reported an insufficient rating for customer satisfaction (5/10).

### 3.7. Correlations Between Device Usage per Day and Modified EQ-5D-5L, FoG-Related Questions and Customer Satisfaction

Two participants reported that they used the device only as needed. Therefore, these participants could not be included in the calculation of the correlations.

None of the correlations between device usage per day and the various domains of the modified EQ-5D-5L, the FoG-related questions, and customer satisfaction were significant (Table 2). Although not significant, a moderate positive correlation was found between device usage per day and ADL (r = 0.452, *p* = 0.108).

## 4. Discussion

This pilot study aimed to determine the user experiences and device usage of the Cue2walk smart cueing device for FoG in people with PD and its impact on perceived health-related QoL, FoG, and daily activities. The participants in this study used the Cue2walk device 9 h per day on average. Positive effects since using the Cue2walk device were predominantly reported on the duration of FoG episodes, falls, mobility, ADL, and self-confidence, while no negative effects were reported. Furthermore, a significant improvement was found for overall health-related QoL at the moment of filling out the survey compared to before using the Cue2walk device, and customer satisfaction was rated with a 7.8 on a 0–10 scale.

For the VAS score for overall health-related QoL in the EQ-5D-5L, the minimal clinically important difference (MCID) has not been determined in people with PD, as far as we know. However, the improvement found in this study exceeds the estimates of the MCID for the EQ-5D-5L VAS score of many other disease populations, such as stroke (anchor-based MCID = 0.86) and cancer (anchor-based MCID = 0.50) [21,22,23,24,25]. It is therefore likely that the improvement that we found for overall health-related QoL in this study is clinically relevant.

A majority of the respondents in this study reported experiencing a shorter duration of FoG episodes since using the Cue2walk device. This is in line with the study by Bächlin et al. (2010), in which a majority of the participants also reported to experience shorter FoG episodes when using a smart cueing device [18]. Subjective measures in FoG can have low reliability [26] and are prone to bias [27], so objective measures are needed. In previous studies in which the effects of smart cueing on the duration of FoG episodes were determined objectively during walking tasks, both neutral and positive results were found [16,28]. In the first study, 20 participants completed the 2 min walk test and a gait circuit. When using a (visual) smart cueing device, FoG duration did not differ compared to receiving no cueing. However, these neutral results were likely due to low statistical power [16]. The second study found an average reduction of 27% in FoG duration from no cueing to (vibratory) smart cueing in six participants that completed a gait circuit [28]. Smart cueing therefore seems to have a positive effect on the duration of FoG episodes, but to elucidate the effects of the Cue2walk device on this domain, it is important to test the Cue2walk device with objective measures, e.g., video annotation during a walking task, standardized clinical tests, and/or analysis of accelerometer data. We are currently setting up a study in which the latter forms a part of it [29].

In accordance with the previous literature on the effects of smart cueing, a majority of the respondents in the present study reported improved mobility and health-related QoL [30]. To our knowledge, no studies have been performed on the effect of smart cueing on the other domains investigated in this study. However, positive effects on falls [31,32] and ADL [33,34] were also found in previous research using conventional cueing. The present study therefore suggests that the positive effects of conventional cueing methods on these domains also apply when smart cueing is used.

One respondent reported to have experienced no impact since using the Cue2walk device on any of the domains of the modified EQ-5D-5L, including overall health-related QoL, nor on the three FoG-related questions, and rated customer satisfaction a 5 on a 0–10 scale. Responsiveness to external cueing can be highly individual [35,36], and different types of cueing may have different effects on gait [37]. Although a thorough assessment takes place as to whether a new user of the Cue2walk device responds well to external cueing, it could be that the cueing functionalities the Cue2walk device offers were not helpful for this respondent’s specific walking problems in the long-term and that other compensation strategies or types of cueing will be more beneficial for this respondent.

Inherent to its design, this pilot study has several limitations. First of all, the survey is partly retrospective and is therefore subject to recall bias, since average device possession was 6 months. Also, this study has a small sample size and did not include a control group or double baseline measurement. Furthermore, it is possible that mainly people who experienced a positive impact since using the device have responded to the survey. For the eight people that were invited but did not fill out the survey, it is unknown whether this was the case or that they did not fill out the survey because of other reasons, e.g., they did not see the mail or did not receive it well. Additionally, the survey was very concise, important aspects of FoG, such as duration, frequency, and circumstance of the FoG episodes, have not been measured in detail or not at all, and disease severity and medication intake have not been determined. Moreover, our modification to the EQ-5D-5L made the questionnaire susceptible to recall bias and we have not assessed the reliability or validity of our modified version. The interpretation of these results should therefore take into account potential methodological constraints. Lastly, all correlations made should be interpreted with high caution, since missing responses were interpolated, the responses were divided amongst three categories (neutral, positive, and very positive), and the sample size is small. Therefore, the non-significant correlations could also be due to insufficient statistical power rather than the absence of an effect. Taking into account the aforementioned limitations, it is yet to unravel whether the positive results found in this study would also be found in the entire population of people with PD experiencing FoG.

In conclusion, this pilot study suggests that the Cue2walk device is a valuable tool for effectively managing FoG in daily life and enhancing QoL in a subgroup of people with PD. The results justify further research on the effects of using the Cue2walk device, in which larger populations, an extended follow-up, a larger test battery with validated questionnaires (e.g., Parkinson’s Disease Questionnaire-39, Nottingham Extended ADL-index, and New Freezing of Gait Questionnaire), and physical measurements (e.g., Timed Up and Go test and 6 min walk test with and without wearing a Cue2walk device) are recommended to validate these findings and assess the long-term efficacy and cost-effectiveness of the device.

## Figures and Tables

**Table 1:** Participant characteristics.

Participant Characteristic	Mean (Standard Deviation) or n
Age (years)	74 (7)
Sex (M/F)	12/4
Disease duration (years)	10 (7)
Device usage total (months)	6 (4)
Device usage per day (h/day)	9 (4) (n = 14) As needed (n = 2)

**Table 2:** Correlations between device usage and the modified EQ-5D-5L, FoG-related questions, and customer satisfaction.

Domain	Device Usage per Day	
	r	*p*
Mobility	0.320	0.257
Self-care	0.129	0.660
ADL	0.452	0.108
Pain/discomfort	0.329	0.272
Anxious/depressed	0.089	0.778
Duration of FoG episode	0.358	0.217
Falls	0.274	0.346
Self-confidence	0.188	0.520
Customer satisfaction	0.331	0.245

ADL, activities of daily living; FoG, freezing of gait.

## Data Availability

The data presented in this study are available on request from the corresponding author.

## Abbreviations

The following abbreviations are used in this manuscript:

ADL	Activities of daily living
FoG	Freezing of gait
EQ-5D-5L	EuroQol 5 dimensions 5 level survey
MCID	Minimal clinically important difference
PD	Parkinson’s disease
QoL	Quality of life
VAS	Visual analogue scale

## Appendix A. Online Survey

Demographics

- Are you: Male/Female
- What is your age?
- How long ago (in years) were you diagnosed with Parkinson’s disease?
- What medication do you use for your Parkinson’s disease and in what amount per day?

Cue2walk device usage

- How long (in months) have you been using the Cue2walk device?
- How many hours per day do you wear the Cue2walk device?

Modified EQ-5D-5L

- Have you experienced any changes in your mobility since using the Cue2walk device?
  I experience
  ○ much less walking problems;
  ○ less walking problems;
  ○ no more or less walking problems;
  ○ more walking problems;
  ○ much more walking problems.
- Have you experienced any changes in your self-care since using the Cue2walk device?
  I experience
  ○ much less problems with self-care;
  ○ less problems with self-care;
  ○ no more or less problems with self-care;
  ○ more problems with self-care;
  ○ much more problems with self-care.
- Have you experienced any changes in the way you perform your usual activities since using the Cue2walk device?
  I experience
  ○ much less problems with daily activities;
  ○ less problems with daily activities;
  ○ no more or less problems with daily activities;
  ○ more problems with daily activities;
  ○ much more problems with daily activities.
- Have you experienced any changes in your pain and/or discomfort since using the Cue2walk device?
  I experience
  ○ much less pain or discomfort;
  ○ less pain or discomfort;
  ○ no more or less pain or discomfort;
  ○ more pain or discomfort;
  ○ much more pain or discomfort.
- Have you experienced any changes in your anxiety and/or depression since using the Cue2walk device?
  I feel
  ○ much less anxious and/or depressed;
  ○ less anxious and/or depressed;
  ○ no more or less anxious and/or depressed;
  ○ more anxious and/or depressed;
  ○ much more anxious and/or depressed.
- We would like to know how good or bad your health was BEFORE using the Cue2walk device. This scale is numbered from 0 to 10: 10 means the best health you can imagine; 0 means the worst health you can imagine.
- We would like to know how good or bad your health is TODAY. This scale is numbered from 0 to 10: 10 means the best health you can imagine; 0 means the worst health you can imagine.

Additional FoG-related questions to the modified EQ-5D-5L

- To what extent does the use of the Cue2walk device influence the duration of a freezing of gait episode?
  The duration of a freezing of gait episode is
  ○ much shorter;
  ○ shorter;
  ○ not shorter or longer;
  ○ longer;
  ○ much longer.
- To what extent does the use of the Cue2walk device influence the number of falls?
  I experience
  ○ much less falls;
  ○ less falls;
  ○ no more or less falls;
  ○ more falls;
  ○ much more falls.
- To what extent does the use of the Cue2walk device influence your self-confidence?
  I feel
  ○ much more confident;
  ○ more confident;
  ○ not more or less confident;
  ○ less confident;
  ○ much less confident.

Customer satisfaction

- How likely are you to recommend the Cue2walk device to someone else with Parkinson’s disease experiencing freezing of gait on a scale of 0 to 10?
